# Metals in the drinking water of First Nations across Canada

**DOI:** 10.17269/s41997-021-00497-5

**Published:** 2021-06-28

**Authors:** Harold Schwartz, Lesya Marushka, Hing Man Chan, Malek Batal, Tonio Sadik, Amy Ing, Karen Fediuk, Constantine Tikhonov

**Affiliations:** 1 First Nations and Inuit Health Branch, Indigenous Services Canada, Ottawa, Canada; 2 Department of Biology, University of Ottawa, 30 Marie Curie, Ottawa, ON K1N 6N5 Canada; 3 Département de Nutrition, Faculté de Médecine, Université de Montréal, Pavillon Liliane de Stewart, C.P. 6128, succ. Centre-Ville, Montréal, QC H3T 1A8 Canada; 4 Centre de recherche en santé publique de l’Université de Montréal et du CIUSS du Centre-sud-de-l’Île-de-Montréal (CReSP), 7101 Avenue du Parc, Montréal, QC H3N 1X7 Canada; 5 Assembly of First Nations, 55 Metcalfe Street, Suite 1600, Ottawa, ON K1P 6L5 Canada

**Keywords:** Metals, Drinking water, First Nations, Lead, Aluminum, Manganese, Métaux, eau potable, Premières Nations, plomb, aluminium, manganèse

## Abstract

**Objectives:**

The First Nations Food, Nutrition and Environment Study (FNFNES), a community-based participatory research project, measured the levels of metals of health concern and the levels of metals that have operational guidance (OG) and aesthetic objectives (AO) in drinking water of First Nations (FN) south of the 60^th^ parallel.

**Methods:**

Both stagnant (first draw) and flushed tap water samples were collected from participating households in 91 FN representing 11 ecozones. The concentrations of metals were quantified and compared to Health Canada’s Guidelines for Canadian Drinking Water Quality (GCDWQ).

**Results:**

In total, 1516 FN households participated in this study component. Exceedances of the 2019 GCDWQ for the health-based maximum acceptable concentration (MAC) were found for five metals: lead 8.4% of households (first draw), manganese 4.0%, uranium 1.6%, aluminum 1.3%, and copper 0.2% (flushed). Flushing taps resulted in a decrease to 0.7% of households exceeding the lead MAC. Exceedances of the current OG for aluminum were found in 14.2% and the exceedances of the proposed OG were found in 18.1% of households (flushed). Exceedances of the AO (flushed) were as follows: manganese 12.8%, sodium 5.1%, iron 3.5%, and copper 0.4%. Results of the study were compared to provincial surveys where the data were available. Taste and colour were reported to be the main reasons for limiting the use of tap water.

**Conclusion:**

Overall, the quality of drinking water with respect to trace metals of human health concern is satisfactory. However, elevated lead levels were found in some FN communities. Until appropriate action can take place, it was recommended to flush the water before use to reduce the lead levels.

## Résumé

**Objectifs:**

L’Étude sur l’alimentation, la nutrition et l’environnement chez les Premières Nations (EANEPN), un projet de recherche participative communautaire, a mesuré la concentration de métaux préoccupants pour la santé et ceux ayant une directive opérationnelle et des objectifs esthétiques dans l’eau potable des Premières Nations (PN) au sud du 60^e^ parallèle.

**Méthodes:**

Des échantillons d’eau stagnante (premier écoulement) ainsi que ceux d’eau après rinçage des robinets ont été prélevés auprès de ménages participants, dans 91 PN, représentant 11 écozones. Les concentrations en métaux ont été quantifiées et comparées à celles des recommandations pour la qualité de l’eau potable au Canada (RQEPC) de Santé Canada.

**Résultats:**

Au total, 1 516 ménages des PN ont participé à ce volet de l’étude. La concentration maximale acceptable (CMA) pour la santé selon les RQEPC de 2019 a été dépassée pour cinq métaux: le plomb, dans 8,4 % des ménages (premier écoulement); le manganèse dans 4,0 % des ménages; l’uranium dans 1,6 % des ménages; l’aluminium, dans 1,3 % des ménages; et le cuivre, dans 0,2 % des ménages (après le rinçage des robinets). Le rinçage des robinets a abouti à une baisse de la concentration en plomb de 0,7% au niveau des ménages dépassant la CMA. La concentration en aluminium a dépassé la valeur de la directive opérationnelle actuelle dans 14,2 % des ménages. Elle a été dépassée et la valeur opérationnelle recommandée dans 18,1 % des ménages (après le rinçage des robinets). Pour les objectifs esthétiques (après le rinçage des robinets), les dépassements des valeurs de concentration des métaux étaient les suivants : le manganèse, dans 12,8 % des ménages; le sodium, dans 5,1 % des ménages; le fer, dans 3,5 % des ménages; et le cuivre, dans 0,4 % des ménages. Les résultats de l’étude ont été comparés aux sondages provinciaux où les données provinciales étaient disponibles. Le goût et la couleur seraient les principales raisons invoquées pour la réduction de l’utilisation de l’eau du robinet.

**Conclusion:**

Dans l’ensemble, la qualité de l’eau potable est satisfaisante en ce qui concerne les niveaux de traces de métaux présentant un risque pour la santé humaine. Toutefois, des niveaux de plomb élevés ont été constatés dans certaines collectivités des PN. En attendant que des mesures appropriées soient prises, il a été recommandé de rincer le robinet (faire couler l’eau) avant d’utiliser l’eau pour réduire les niveaux de plomb.

## Introduction

The quality of drinking water is regulated by Health Canada’s Guidelines for Canadian Drinking Water Quality (GCDWQ) (Health Canada 2019b). The metal guidelines are established based on current scientific knowledge about the effects of metals on human health (e.g., maximum acceptable concentration (MAC)); physical parameters (e.g., taste, smell, or colour) that may affect consumer acceptance of drinking water (such as iron, zinc, and sodium), otherwise known as aesthetic objective (AO); and operational guideline values (OG) established for parameters that may affect treatment processes or impair drinking water distribution system (such as for aluminum) (Health Canada 2019b)*.*

Metals are trace elements that are naturally occurring in different environment media (Tchounwou et al. 2012). Some metals, for example lead, copper, and antimony, can also release into drinking water from the plumbing pipes in the water distribution systems (Health Canada 2009; Chowdhury et al. 2016). Some of these elements, including zinc, copper, iron, and manganese, are essential nutrients that are required in small amounts to maintain the metabolism of the human body (Mehri 2020; Zoroddu et al. 2019). Inadequate intake of these micronutrients may lead to a variety of deficiency syndromes (Mehri 2020; Zoroddu et al. 2019). However, if ingested at higher concentrations, these chemicals can be toxic (Jamshaid et al. 2018). Other elements, such as mercury, cadmium, arsenic, and lead, have no known biological functions and are toxic even at low concentrations (Tchounwou et al. 2012).

Metals can also enter the environment through anthropogenic sources, such as mining and smelting operations, paper processing, waste disposal, and domestic and agricultural use (Bradl 2005; Tchounwou et al. 2012). They can contaminate the surface water through effluents or runoff or leach into the soil and contaminate the groundwater. In Canada, about 30% of the population relies on groundwater for drinking (ECCC 2013). Long-term exposure to heavy metals is associated with various adverse health effects, including cancer (Tchounwou et al. 2012; Jamshaid et al. 2018). The distribution and health effects of selected metals are described below.

Arsenic (As) can be present in different chemical forms with varying toxicities. Inorganic arsenic is one of the most toxic forms and is naturally found in groundwater. Arsenic compounds are also used commercially and may enter into drinking water sources from industrial effluents (Health Canada 2006). Acute exposure to arsenic causes abdominal pain, metallic and garlic taste in the mouth, vomiting, and watery diarrhea (Health Canada 2006). Chronic exposure to elevated levels of inorganic arsenic is known to cause several types of cancer, such as skin, bladder, liver, kidney, and lung. Arsenic exposure is also associated with an increased risk of diabetes, heart diseases, and peripheral vascular diseases (Health Canada 2006).

Lead (Pb) is usually present in drinking water due to leaching from lead-containing service lines, lead solder, and brass fittings, particularly in corrosive waters. Since lead was extensively used in drinking water distribution and plumbing systems prior to 1986, elevated lead concentrations are more likely to be found in older homes (Health Canada 2019d). Infants and children are most susceptible to lead toxicity, including irreversible neurodevelopmental effects, impaired cognitive development, and learning and behaviour problems. In adults, chronic lead exposure is associated with reproductive and renal disorders and cardiovascular diseases (Health Canada 2019d). There is no population threshold for adverse neurodevelopmental effects. Therefore, every effort should be made to maintain lead levels in drinking water as low as reasonably achievable (Health Canada 2019d).

Uranium (U) is a radioactive metal that occurs naturally through erosion and weathering of rocks and soils. Therefore, its levels in drinking water vary depending on geological formations near the source water (Health Canada, 2017b, 2019e). Chronic exposure to uranium is associated with kidney effects (Health Canada, 2017b, 2019e).

Aluminum (Al) is widely used as a coagulant in drinking water treatment. An OG value of 50 μg/L was proposed for aluminum to minimize the potential accumulation and release of aluminum in the distribution systems (Health Canada 2019a). Several epidemiological studies have reported positive associations between chronic exposure to aluminum and neurological disorders, including Alzheimer’s disease (Health Canada 2019a). However, there is still limited evidence to state a causal relationship between aluminum exposure and neurological diseases (Health Canada 2019a).

Selenium (Se) is an essential trace element in human nutrition that is involved in the regulation of thyroid hormones and antioxidants. Exposure to relatively high levels of selenium from drinking water may result in selenosis symptoms characterized by hair loss, nail anomalies or loss, skin anomalies, and disturbances of the nervous system (Health Canada 2014).

Copper, iron, manganese, zinc, and sodium are essential elements and the primary concerns regarding their presence in drinking water are their effect on the taste, odour, and colour, as well as the staining of laundry and plumbing fixtures. No adverse health effects are associated with levels of these metals typically found in drinking water, within the established AOs (Health Canada 2019b). However, there is increasing evidence that manganese is associated with neurotoxicity ((Dobson et al. 2004; Erikson & Aschner, 2019). In fact, several studies have reported an association between exposure to elevated levels of manganese via drinking water and neuropsychological disorders in infants and children, such as behaviour problems, lower IQ, and speech and memory difficulties (Dobson et al. 2004; Erikson & Aschner, 2019). Short-term exposure to high doses of copper may cause gastrointestinal symptoms, such as nausea, epigastric pain, vomiting, or diarrhea. Chronic copper toxicity is usually observed in patients with Wilson’s disease, the rare inherent disease characterized by excessive accumulation of copper in various body tissues, such as liver, brain, and corneas of the eyes, and may lead to liver and renal failure (Health Canada 2018).

The concentrations of a variety of metals in drinking water were measured in all provinces and territories for the National Survey of Disinfection By-Products and Selected Drinking Water Contaminants in Canadian Drinking Water 2009–2010 (Health Canada 2019b). The water samples were collected from 65 water treatment systems across Canada. Although the results were not statistically representative of the Canadian population, they provided a snapshot of metal concentrations in treated and distributed water at the national and regional levels. This survey, however, did not include drinking water quality data relating to First Nations communities; thus, there have been ongoing concerns about the need for quality research on drinking water and its health effects on First Nations (Bradford et al. 2016). Therefore, the goal of the First Nations Food, Nutrition and Environment Study’s (FNFNES) drinking water component was to determine the chemical safety of tap water in First Nations by quantifying the metals in drinking water samples collected from randomly selected houses in First Nations across Canada and comparing results to the drinking guideline levels established by Health Canada.

## Methods

### Study design

Detailed information on the FNFNES design and methods is presented in Chan et al. (2021) in this issue. The FNFNES was designed to assess traditional food consumption, the exposure to environmental contaminants, total diets, and the chemical safety of drinking water supplies of First Nations people living on reserves, south of the 60^th^ parallel across Canada. The study, implemented over 10 years, is representative of First Nations in the eight Assembly of First Nation regions south of the 60^th^ parallel. Each FNFNES region was comprised of the province (British Columbia, Alberta, Saskatchewan, Manitoba, Ontario, and Quebec-Labrador) except the Atlantic Region where Nova Scotia, New Brunswick, Prince Edward Island, and Newfoundland together were categorized as the Atlantic Region.

First Nations communities were randomly selected using a 3-stage sampling strategy: the regions, the communities, and the households. The sampling strategy incorporated an ecosystem framework comprised of 11 ecozones to ensure that the results were representative at the regional and ecozone levels. In each household, one adult who self-identified as a First Nation person living on-reserve, aged 19 years or older, and whose birthday was the next (among the eligible adults in the household), was asked to participate in the survey.

In total, 91 First Nation communities agreed to participate in the metals in drinking water component of the FNFNES. The drinking water component aimed to collect tap water samples from 20 out of approximately 100 participating households in every participating First Nation. Among the randomly selected participants who completed a household interview, the first 20 households that agreed to participate in the drinking water component were selected for water sampling. Maps, provided by local First Nation leaders, were used to confirm that the 20 houses were representative of the water distribution systems (i.e., at the end of the pipelines and miscellaneous points within the system) used in the First Nations communities. Additionally, if a household was using an alternate source of drinking water that was not part of the community water supply system, such as a well, nearby spring, or a trucked water source, they were also sampled. The use of river or lake water as a drinking water source in some communities was necessitated by drinking water advisories and lack of accessibility to bottled water. Where the communities used bottled water as a supplemental water source, a sample of bottled water was also analyzed. These alternate water sources were not included in this analysis.

At each home selected to participate in this study component, two tap water samples were collected by a trained First Nation community member: the first draw sample was collected by the householder after the water had been sitting stagnant in the pipes for a minimum of 4 h. Later that same day, a flushed sample was collected after running the water for 5 min. Health Canada recommends a 6-h stagnation time for sampling metals in drinking water (Health Canada 2009). Due to the large number of people living in most First Nation homes and after discussion with First Nations on what was practicably achievable, this study limited the stagnation time for drinking water sampling to 4 h. Twenty-five percent of the flushed samples were run in duplicate for quality assurance and quality control purposes. Duplicate samples were collected on the same day immediately after flushed sample collection. The water was flushed for a max of 2 min in between the samples.

Information about water sources (community water treatment system, well), delivery to the household (piped, cistern, etc.), and age and type (metal, plastic) of plumbing in the house had previously been recorded as part of the household questionnaire. Also, community water operators were interviewed in person about aspects of the community water system (water source, age, households served, and types of delivery, chemicals used in the treatment, and holding tanks). The interviews took place during the fall months from 2008 to 2016.

### Tap water analysis

Water samples were sent for analysis to Cantest/Maxxam in Vancouver from 2008 to 2011 (British Columbia, Manitoba, and Ontario (year 1)) and ALS Global, in Waterloo, Ontario, in 2012 to 2016 (Ontario (year 2), Alberta, the Atlantic, Saskatchewan, and Quebec). Inductively coupled argon plasma mass spectroscopy (ICP/MS) was used to perform all analyses for the elements requested (using methodology based upon EPA Method # 200.8). When metal was present in a sample at a high concentration, a dilution was required to reduce its concentration to within the calibrated range of the test method or to prevent damage to sensitive instruments. The detection limit was then increased to reflect the dilution factor. For multi-parameter tests, detection limits were increased for all parameters within the test, whether they were detected or not. In this study, all sample results are reported as micrograms per litre (μg/L) (Chan et al. 2018). The detection limits for the metals are reported in Table 2.

The FNFNES monitored ten metals of public health concern that have MAC under Health Canada’s GCDWQ (Health Canada 2017a): antimony, arsenic, barium, boron, cadmium, chromium, lead, mercury, selenium, and uranium. In addition, six metals that have AO or OG (aluminum, copper, iron, manganese, sodium, and zinc) were quantified in drinking water (Table 3).

### Comparison with drinking water guidelines

In June 2019, Health Canada revised the GCDWQ for lead, copper, manganese, and aluminum (Health Canada 2019b). The MAC for lead was reduced from 10 μg/L to 5 μg/L (Health Canada 2019d). For copper, in addition to its guidance for an AO (1000 μg/L), a MAC of 2000 μg/L was established (Health Canada 2018). A MAC of 120 μg/L was set for manganese while its AO guidance value was revised from 50 μg/L to 20 μg/L (Health Canada 2019c). In August 2019, Health Canada proposed to reduce the OG for aluminum from 100 μg/L to 50 μg/L and proposed a MAC of 2900 μg/L in the Guideline Technical Document for Public Consultation (Health Canada 2019a).

This paper evaluated the exceedances based on the previous guidelines (Health Canada 2017a) since they were reported to the First Nation communities as well as on the 2019 guidelines (Health Canada 2019b). The revised and proposed MAC values are shown in Table 3. Exceedances were considered if concentrations in *flushed water samples* were above the respective guideline values for all metals except lead (Health Canada 2017a, 2019d). Based on the revised guideline, lead exceedances are now considered based on *first draw samples* (Health Canada 2019d).

## Results and discussion

### Availability and use at the household level

In total, 91 First Nation communities participated in the drinking water component of the FNFNES. A total of 1516 households took part in the drinking water portion of the study or on average 16.7 households per community (Table 1). Table 12 in Appendix 4 shows the sample collection by year and ecozone with the number of the first draw, flushed samples, and duplicate samples collected (Appendix 4). The participation rate for the metals in drinking water component was 80.1%.

**Table 1:** First Nation communities participating in the FNFNES drinking water component by region

Year	Region	No. of communities	No. of households	No. of first draw samples	No. of flushed samples	No. of samples
2008–2009	British Columbia	20	314	300	300	600
2010	Manitoba	9	143	140	142	282
2011–2012	Ontario	18	334	318	322	640
2013	Alberta	9	108	93	106	199
2014	Atlantic Canada	11	216	216	217	433
2015	Saskatchewan	14	234	228	224	452
2016	Quebec	10	167	160	167	327
	Total	91	1516	1455	1478	2933

**Fig. 1:**
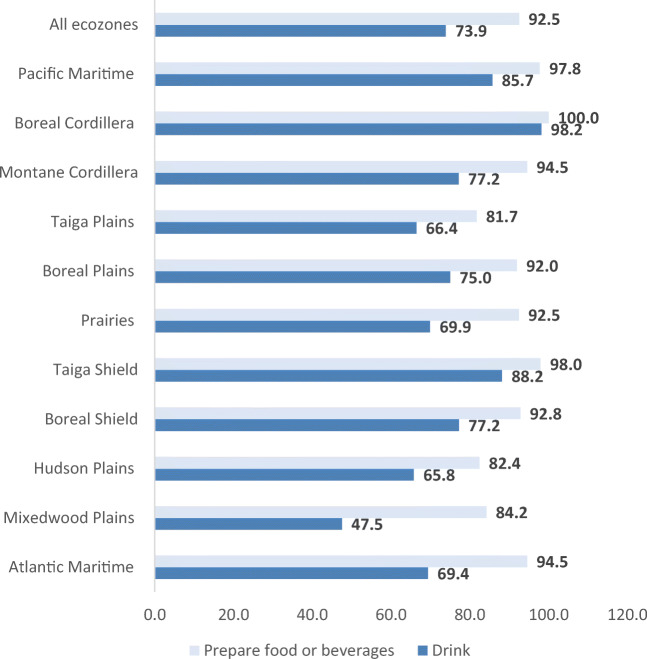
Household tap water use by ecozone (Chan et al., 2019)

Overall, 78% of households participated in the household survey while the participation rates ranged from 68% to 90% across regions (data are not shown). Almost all respondents (99.5%) reported that they had tap or treated drinking water. Among households with tap water, 79% reported receiving tap water from the community’s public water system (71.2% piped, 7.6% trucked in), while 14.8% were on a well or individual water system and 2.2% of households received water through a municipal transfer agreement (between a First Nation and a municipal government for a service, such as the delivery of treated drinking water from the municipal water treatment system by pipe or truck to households on reserve lands). The use of non-treated surface water for drinking was reported by 4% of households, while 0.2% said that they relied on a rainwater cistern. Although almost all households had tap water, only 73.9% stated that they used it for drinking, while 92.5% reported using tap water for cooking purposes. Data on the tap water use by ecozone are presented in Fig. 1 (Appendix 5). The main reason for tap water avoidance for greater than 25% of First Nations was the concern about the taste and colour of the water.

### Metals of public health concern

Table 2 summarizes data on metals concentrations in drinking waters in First Nations by regions. Overall, the mean/median concentrations of lead in water samples were higher in Ontario, Manitoba, and the Atlantic region than in other regions. The levels of aluminum were generally low except in Manitoba with the mean and medial concentrations as high as 1824.5 μg/L and 120 μg/L, respectively. Copper concentrations ranged from 14.1 to 143.6 μg/L across regions. The highest mean and median concentrations of manganese were detected in the Atlantic region (52.0 μg/L and 6.0 μg/L, respectively) followed by Manitoba (47.9 μg/L and 3.4 μg/L, respectively) and Saskatchewan (35.9 μg/L and 2.2 μg/L).

**Table 2:** Metals concentrations in drinking waters in First Nations communities by regions

	Sample size	Detection limit (μg/L)	Mean (μg/L)	Median (μg/L)	75th (μg/L)	95th (μg/L)	Max (μg/L)
British Columbia (2008–2009)							
Lead	300	0.2	1.0	0.6	1.2	3.9	6.3
Uranium	300	0.1	0.5	0.3	0.5	1.9	4.5
Aluminum	300	1	16.6	2.0	9.0	147.3	287.0
Copper	300	0.2	35.8	11.2	33.4	146.3	618.0
Iron	300	10	31.3	19.0	48.0	79.1	527.0
Manganese	300	0.2	5.3	0.4	1.0	24.9	250.0
Sodium	300	10	11,442	3265	13,625	28,625	292,000
Zinc	300	1	23.9	4.0	7.0	72.1	1690.0
Manitoba (2010)							
Lead	140	0.2	2.8	1.0	3.1	11.1	24.7
Uranium	142	0.1	0.8	0.5	0.8	2.7	9.6
Aluminum	142	1	1824.5	120.0	471.8	13,185.0	18,200.0
Copper	142	0.2	143.6	74.2	366.0	1060.0	6540.0
Iron	142	10	48.7	17.0	42.5	149.6	1700.0
Manganese	142	0.2	47.9	3.4	21.3	278.8	321.0
Sodium	142	10	57,235	9090	130,750	217,700	392,000
Zinc	142	1	173.7	13.0	47.3	366.8	5150.0
Ontario (2011–2012)							
Lead	318	0.2	3.4	0.7	2.1	13.7	120.0
Uranium	322	0.1	2.0	<0.1	0.7	20.7	37.1
Aluminum	322	0.1	77.2	20.0	57.0	505.5	1320.0
Copper	322	0.2	68.2	22.3	49.9	268.7	3380.0
Iron	322	10	30.2	<10	32.0	104.9	925.0
Manganese	322	0.2	6.28	1.83	6.40	39.10	96.00
Sodium	322	10	23,500	12,800	22,150	56,260	840,000
Zinc	322	1	22.2	<1	4.5	92.5	1730.0
Alberta (2013)							
Lead	93	0.2	1.2	<0.5	0.7	4.2	45.0
Uranium	106	0.1	<1	<1	<1	1.2	1.5
Aluminum	106	1	72.9	<1	43.0	340.0	566.0
Copper	106	0.2	14.1	5.6	16.6	65.8	120.0
Iron	106	10	124.2	<50	<50	495.0	5500.0
Manganese	106	0.2	11.7	3.0	16.9	51.5	87.5
Sodium	106	10	97,649	53,100	81,700	376,000	544,000
Zinc	106	1	14.4	6.5	12.4	56.8	314.0
Atlantic (2014)							
Lead	216	0.5	3.3	<1	1.2	18.0	107.0
Uranium	217	1	<1	<1	<1	1.1	1.4
Aluminum	217	10	30.0	<10	15.3	124.3	203.0
Copper	217	1	70.9	30.0	85.4	281.3	673.0
Iron	217	50	75.1	<50	66.0	377.5	407.0
Manganese	217	1	52.0	6.0	20.0	449.0	813.0
Sodium	217	500	30,265	21,250	36,325	126,000	132,000
Zinc	217	3	14.5	5.9	11.1	63.2	373.0
Saskatchewan (2015)							
Lead	228	0.1	1.2	0.4	1.1	4.6	43.8
Uranium	224	0.01	2.3	0.03	4.5	12.5	46.2
Aluminum	224	10	21.4	<10	13.0	92.4	196.0
Copper	224	1	42.7	12.5	35.2	195.6	1190.0
Iron	224	50	<50	<50	<50	<50	2790.0
Manganese	224	0.5	35.9	2.2	4.7	107.0	3250.0
Sodium	224	500	65,141	9190	27,700	644,400	698,000
Zinc	224	3	28.1	5.4	11.6	96.3	1220.0
Quebec (2016)							
Lead	160	0.1	1.2	0.5	1.1	3.6	25.3
Uranium	167	0.01	0.5	0.1	0.5	1.9	9.5
Aluminum	167	10	17.6	<10	18.0	64.8	157.0
Copper	167	1	45.6	20.7	54.3	190.4	435.0
Iron	167	50	72.4	<50	<50	38.6	5070.0
Manganese	167	0.5	11.1	0.8	1.6	18.7	929.0
Sodium	167	500	37,590	13,600	16,450	219,000	866,000
Zinc	167	3	44.7	5.2	10.0	324.8	388.0

Estimates on metals concentrations are based on flushed samples for all metals except lead

For lead, all estimates are based on first draw samples (Health Canada 2019d)

Of the 10 metals of health concern tested by this study, only *arsenic*, *lead*, *selenium*, and *uranium* exceeded the GCDWQ. The concentrations of antimony, barium, boron, cadmium, chromium, and mercury were well below the respective MAC values and, therefore, are not presented in this article. For more details on metals in drinking water in First Nations south of the 60^th^ parallel, see the FNFNES Final Report for Eight Assembly of First Nations Regions: Draft Comprehensive Technical Report (Chan et al. 2019).

#### Arsenic

The MAC for total arsenic in drinking water is 10 μg/L (Health Canada 2019b). In First Nations of Canada, elevated levels of arsenic were found in three households in two communities in the Prairies ecozone in the Saskatchewan region. Following a 5-min flush, only one household of the 234 homes participating in the Saskatchewan region exceeded the GCDWQ level of 10 μg/L (Chan et al. 2018).

In the Saskatchewan provincial survey (Health Canada 2006), 97% of the homes were found to have levels less than or equal to 10 μg/L. From 1976 to 2002, arsenic levels were found to be from 0.5 to 105 μg/L. The arsenic levels found in the FNFNES Saskatchewan survey were similar to those found in the provincial survey (Health Canada 2006). Overall, arsenic does not represent a concern in Canadian municipal drinking water systems. The concentration of arsenic in drinking water in areas without natural sources is usually less than 5 μg/L (Health Canada 2006). However, several areas in southern British Columbia, Alberta, Manitoba, and Quebec, northeastern Saskatchewan as well as throughout Nova Scotia, Newfoundland and Labrador, and New Brunswick have elevated levels of arsenic which are defined as “hotspots” (i.e., >10 μg/L). These hotspots are generally due to natural sources. The maximum concentration of 5000 μg/L of arsenic was found in groundwater in Nova Scotia (McGuigan et al. 2010).

#### Lead

The 2019 GCDWQ for lead is 5 μg/L (reduced from 10 μg/L) and is based on concentrations found in the first draw water samples randomly collected during the day (random daytime sampling (RDT)) or after 30 min of stagnation (previously, concentrations were measured in a 5-min flushed sample) (Table 3) (Health Canada 2017a; Health Canada 2019d). The 4-h stagnant sampling in the present study is compared with the 30-min stagnant residential sampling of the most recent Health Canada water sampling procedure (Health Canada 2019d). These two sampling protocols would most likely yield slightly different results. Based on the previous version of the GCDWQ and the values reported to the First Nation communities, only three households (0.2%) exceeded the guideline following a 5-min flush: one household in the Boreal Plains ecozone in Saskatchewan with a value of 22 μg/L (Chan et al. 2018); one household in the Boreal Shield ecozone in Manitoba with a value of 24.7 μg/L (Chan et al. 2012); and one household in the Mixedwood Plains ecozone in Ontario with a value of 12.0 μg/L (Chan et al. 2014). These three exceedances were reported to the First Nations communities based on the lead in drinking water guidelines at that time (Health Canada 2017a). Lead levels above 10 μg/L were also found in 71 first draw water samples (4.7%). Based on the current guideline, an extra 57 first draw samples and 8 flushed samples were within the range of 5 to 9.9 μg/L. This resulted in a total of 128 (or 8.4%) first draw sample and 11 (or 0.7%) flushed sample exceedances (Table 4).

**Table 3:** Guidelines for Canadian drinking water quality for metals measured by FNFNES (Health Canada 2017a, 2019b)

	AO/OG (μg/L), 2017	AO/OG (μg/L), 2019	MAC (μg/L), 2017	MAC (μg/L), 2019
Metals of human health concern				
Antimony	-	-	6	6
Arsenic	-	-	10 (ALARA)	10 (ALARA)
Barium	-	-	1000	1000
Boron	-	-	5000	5000
Cadmium	-	-	5	5
Chromium	-	-	50	50
Lead	-	-	10	5 (ALARA)
Mercury	-	-	1	1
Selenium	-	-	50	50
Uranium	-	-	20 (proposed)	20
Metals with aesthetic objectives and operational guideline values				
Aluminum	<100/200	50 (proposed)	-	2900 (proposed)
Copper	1000	1000	-	2000
Iron	300	300	-	
Manganese	50	20	-	120
Sodium	200,000	200,000	-	-
Zinc	5000	5000	-	-

*MAC*, maximum acceptable concentration (μg/L); *AO*, aesthetic objectives (μg/L); *OG*, operational guideline (μg/L); *ALARA*, as low as reasonably achievable

**Table 4:** Lead concentrations in drinking water by ecozone

Ecozone	No. of first draw samples >10 μg/L	No. of flushed samples >10 μg/L	No. of first draw sample >5–9.9 μg/L	No. of flushed sample >5–9.9 μg/L	Maximum concentration, μg/L
Boreal Cordillera	0	0	1	0	6
Boreal Plains	6	1	8	0	45
Montane Cordillera	0	0	0	0	3.6
Pacific Maritime	3	0	1	0	20.4
Taiga Plains	0	0	2	0	7.9
Taiga Shield	2	0	4	0	11.1
Boreal Shield	38	1	14	6	107
Prairies	2	0	8	0	12.3
Hudson Plains	12	0	4	2	88.9
Mixedwood Plains	8	1	10	0	34.7
Atlantic Maritime	0	0	5	0	8.6
Total	71	3	57	8	107

Previous maximum acceptable concentration, MAC: >10 μg/L (Health Canada 2017a)

Current MAC: >5 μg/L (Health Canada 2019d)

In comparison, data from the Saskatchewan provincial survey indicated that the median lead concentration for 176 samples analyzed was 6.7 μg/L, with concentrations ranging from <0.1 to 60 μg/L (Health Canada 2019d). The province of Ontario collected 37,000 water samples from 2007 to 2008 and ≤3.1% exceeded the guideline of 10 μg/L. In 2009, eight communities were identified for retesting and the Ontario Ministry of the Environment found a range of 0.02 to 1320 μg/L in 3159 samples collected (OMOE 2014). In Portage la Prairie, Manitoba, lead concentrations ranging from 0.1 to 36 μg/L with an average of 0.7 μg/L were detected in 159 tap water samples collected between 2008 and 2009 (Health Canada 2019d).

According to the 2009–2010 Canadian National Drinking Water Survey (CNDWS), which measured lead in flushed water samples at 65 sites across Canada, the average level of lead was 0.9 μg/L during winter (ranging from <0.5 to 8.2 μg/L) and 1.27 μg/L during summer season (ranging from <0.5 to 24 μg/L) (Health Canada 2019d). In a recent cross-Canada study carried out by nine universities and 10 media organizations, including Concordia University’s Institute for Investigative Journalism, 12,000 samples were collected in 32 cities. The results showed that 33% of the samples exceeded 5 μg/L. While water leaving the municipal water treatment plants was found to have low levels of lead, lead service lines, fixtures containing lead, and lead solder in the homes were major sources of lead at the tap (Global News 2019b). In the city of Gatineau, Quebec, this study retested seven homes that had lead values of less than 10 μg/L in the latest municipal testing. Five of the seven homes had lead water levels exceeding 10 μg/L with a maximum level of 140 μg/L and an average level of 30 μg/L (Global News 2019a).

Recently, lead contamination of drinking water has also been found to be a serious problem in the United States in Flint, Michigan (Pieper et al. 2017) and Newark, New Jersey (Viglione 2019). In Flint, Michigan from 2014 to 2017, over 100,000 people were exposed to lead levels as high as 13,200 μg/L (Pieper et al. 2017, 2018). In 2019 in Newark, New Jersey, over 15,000 households were found to have tap water lead levels above the 15 μg/L US Environmental Protection Agency lead in drinking water guideline (Viglione 2019). In both of these crises, lead was found to be leaching from old lead pipes.

#### Uranium

The MAC for uranium in drinking water is 20 μg/L (Health Canada 2019e) (Table 3). In total, 24 households located in three communities had uranium levels above the guideline value in the first draw samples and flushed samples which represent 1.6% (Table 5). Two households in one community in the Prairies ecozone in Saskatchewan had uranium levels above the 20 μg/L guideline. After a 5-min flush, the uranium levels remained elevated with levels ranging from 28 to 46 μg/L. Also, 22 households in two communities in the Boreal Shield ecozone in Ontario had elevated uranium levels in the first draw and flushed water samples ranging from 20 to 58 μg/L.

**Table 5:** Uranium concentrations in drinking water by ecozone

Ecozone	No. of first draw samples >20 μg/L	No. of flushed samples >20 μg/L	Maximum concentration, μg/L
Boreal Cordillera	0	0	0.4
Boreal Plains	0	0	13
Montane Cordillera	0	0	10.3
Pacific Maritime	0	0	0.6
Taiga Plains	0	0	0.8
Taiga Shield	0	0	2.2
Boreal Shield	22	22	58
Prairies	2	2	46
Hudson Plains	0	0	0.1
Mixedwood Plains	0	0	4.0
Atlantic Maritime	0	0	9.6
Total	24	24	58

Maximum acceptable concentration, MAC: >20 μg/L (Health Canada 2019b)

The province of Saskatchewan tested 3910 water samples over 10 years and found 253 of 3910 or 6.5% of the samples with uranium levels greater than 20 μg/L with an average of 5.5 μg/L and a maximum of 183 μg/L (Health Canada 2019e). Ontario monitored uranium levels in drinking water over 11 years (2004 to 2014). The average uranium level was 0.6 μg/L with a maximum of 17.5 μg/L (Health Canada 2019e). The 2009–2010 CNDWS had an average uranium level of 0.52 μg/L (*n*=646), ranging from below the detection limit (<0.1 μg/L) to 32 μg/L (Health Canada 2019e).

#### Selenium

One household in one Prairies community in Saskatchewan had an elevated selenium level in the first draw sample of 79 μg/L. The flushed sample had a level of 76 μg/L, which is above the selenium MAC of 50 μg/L (Health Canada 2014).

According to the Saskatchewan Department of the Environment and Resource Management, the majority of selenium in drinking water samples collected between 2001 and 2011 were below the detection limit of 1 μg/L. However, Saskatchewan was found to have higher levels of selenium compared with other provinces and territories. No selenium levels were found above the guideline; however, the selenium concentrations above 10 μg/L were detected in 1.3% of Saskatchewan water samples (Health Canada 2014).

As a part of the 2009–2010 CNDWS, the levels of selenium in drinking water were tested at 65 sites across Canadian provinces and territories. All samples were below the detection limit of 2 μg/L. However, these values did not account for selenium that may leach from plumbing materials (Health Canada 2014).

### Metals with AOs and an OG

Of the six metals with AO and OG tested by this study, exceedances were found for all metals. All exceedances were communicated with the respective Chief and Council, environmental public health officers of Indigenous Services Canada, and householders.

#### Aluminum

For aluminum, an OG was reduced from 100 μg/L to 50 μg/L and a MAC of 2900 μg/L was proposed by Health Canada (Health Canada 2019a) as shown in Table 3. Therefore, the evaluation of the FNFNES results on aluminum levels in drinking water samples takes into consideration both the existing and proposed guidelines. The participating First Nation communities received their results based on an OG of 100 μg/L.

Twenty households in one Manitoba Boreal Shield community had first draw aluminum in drinking water samples ranging from 6680 to 33,100 μg/L. The flushed samples had aluminum values from 7120 to 18,200 μg/L. All 20 samples exceeded the proposed MAC of 2900 μg/L (Table 6). The elevated levels arose from problems at the water treatment plants in this community. Resampling of the water treatment plant 2 months later found acceptable aluminum levels.

**Table 6:** Aluminum concentrations (μg/L) in drinking water by ecozone

Ecozone	No. of first draw samples >2900	No. of flushed samples >2900	No. of first draw samples >100–2899	No. of flushed samples >100–2899	No. of first draw samples >50–99	No. of flushed samples >50–99	Maximum concentration, μg/L
Boreal Cordillera	0	0	0	0	0	0	6
Boreal Plains	0	0	40	42	6	2	621
Montane Cordillera	0	0	10	11	0	0	287
Pacific Maritime	0	0	0	0	0	0	37
Taiga Plains	0	0	0	0	0	0	40
Taiga Shield	0	0	15	15	0	0	1060
Boreal Shield	20	20	36	58	28	38	33,100
Prairies	0	0	15	16	3	2	290
Hudson Plains	0	0	21	21	2	1	1920
Mixedwood Plains	0	0	12	11	16	14	596
Atlantic Maritime	0	0	18	21	8	2	543
Total	20	20	167	195	63	59	33,100

Proposed maximum acceptable concentration, MAC: >2900 μg/L (Health Canada 2019a)

Current operational guideline, OG: >100 μg/L (Health Canada 2019b)

Proposed OG: >50 μg/L (Health Canada 2019a)

For comparison, based on Manitoba provincial data, aluminum was detected in 396 of 443 treated surface water samples collected from 2012 to 2017 with a maximum of 7970 μg/L (Health Canada 2019a).

Based on the existing OG of 100 μg/L, there were 187 exceedances in first draw samples and 215 exceedances in flushed samples in the Boreal Plains, Montane Cordillera, Taiga Shield, Boreal Shield, Prairies, Hudson Plains, Mixedwood Plains, and Atlantic Maritime (Table 6). The proposed OG of 50 μg/L resulted in a further 63 first draw and 59 flushed water samples exceedances (Table 6). Overall, 14.2% (215 of 1516) of flushed water samples exceeded the existing OG (e.g., 100 μg/L) and 18.1% (274 of 1516) of households exceeded the proposed aluminum guideline of 50 μg/L. Thus, the adoption of the proposed OG increases the operational guidance exceedances by 3.9%.

For comparison, data from the CNDWS (2009–2010) show that the average aluminum concentrations in source water ranged from 10 to 462 μg/L during summer and from 12 to 357 μg/L during winter seasons (Health Canada 2019a). The FNFNES aluminum exceedances by ecozone are presented in Appendix 1 and summarized in Table 6. This information is also published in Chan et al. (2019).

#### Copper

In 2018, Health Canada revised copper drinking water guidelines and established a MAC of 2000 μg/L. The AO remains at 1000 μg/L (Health Canada 2018). The FNFNES result evaluation includes a comparison with both the AO and the new MAC. The participating First Nation communities received their results based on the AO of 1000 μg/L that was the standard during the study. The FNFNES copper exceedances by ecozone are summarized in Table 7.

**Table 7:** Copper concentrations in drinking water by ecozone

Ecozone	No. of first draw samples >2000 μg/L	No. of flushed samples >2000 μg/L	No. of first draw samples >1000–1999 μg/L	No. of flushed samples >1000–1999 μg/L	Maximum concentration, μg/L
Boreal Cordillera	0	0	0	0	602
Boreal Plains	1	0	8	0	5130
Montane Cordillera	1	0	1	0	2200
Pacific Maritime	4	0	9	0	2930
Taiga Plains	0	0	0	0	337
Taiga Shield	0	0	2	0	1270
Boreal Shield	5	2	21	0	6540
Prairies	0	0	2	1	1890
Hudson Plains	3	0	5	0	3460
Mixedwood Plains	1	1	4	2	5850
Atlantic Maritime	0	0	3	0	1570
Total	15	3	55	3	6540

Proposed maximum acceptable concentration, MAC: >2000 μg/L (Health Canada 2019b)

Aesthetic objectives, AO: >1000 μg/L (Health Canada 2019b)

In total, 15 households in the Boreal Plains, Montane Cordillera, Pacific Maritime, Boreal Shield, Hudson Plains, and Mixedwood Plains ecozones had first draw samples that exceeded 2000 μg/L and three households in the Boreal Shield and Mixedwood Plains ecozones had flushed samples copper levels above the proposed MAC. Thus, flushing water removed copper exceedances by 80% (12 of 15 households).

Regarding the copper AO, a total of 70 first draw samples and 6 flushed samples exceeded 1000 μg/L. Overall, for samples with first draw copper levels in the range between 1000 and 1999 μg/L, flushing for 5 min results in more than 95% (52 of 55) of the flushed samples being below the AO. For comparison, the province of Saskatchewan collected 2423 samples for copper in drinking water from 2005 to 2015 and found a maximum of 3900 μg/L (Health Canada 2018). Overall, the copper values found in the FNFNES were similar to those reported by the provinces (Health Canada 2018). The FNFNES copper exceedances by ecozone are described in Appendix 2 and Table 7 and were published elsewhere (Chan et al. 2019).

#### Manganese

In 2019, Health Canada established a MAC of 120 μg/L for manganese in drinking water (Health Canada 2019c). In addition, the AO was revised from 50 μg/L to 20 μg/L. The results were reported to First Nations based on an AO of 50 μg/L. The manganese in water results are reported here using the new MAC as well as both the previous AO of 50 μg/L and the revised AO of 20 μg/L (Table 8).

**Table 8:** Manganese concentrations in drinking water by ecozone

Ecozone	No. of first draw samples >120 μg/L	No. of flushed samples >120 μg/L	No. of first draw samples >50–119 μg/L	No. of flushed samples >50–119 μg/L	No. of first draw samples >20–49 μg/L	No. of flushed samples >20–49 μg/L	Maximum concentration, μg/L
Boreal Cordillera	0	0	1	1	4	5	69.8
Boreal Plains	2	3	10	9	12	16	191
Montane Cordillera	2	2	2	1	1	4	250
Pacific Maritime	0	0	0	0	1	1	44.4
Taiga Plains	0	0	0	0	1	0	20.6
Taiga Shield	2	6	5	8	7	3	142
Boreal Shield	20	20	0	1	2	4	444
Prairies	5	5	9	11	17	20	1530
Hudson Plains	0	0	0	5	2	4	61.5
Mixedwood Plains	1	1	4	5	10	6	370
Atlantic Maritime	23	23	10	8	17	22	975
Total	55	60	41	49	74	85	1530

Current maximum acceptable concentration, MAC: 120 μg/L (Health Canada 2019b)

Previous aesthetic objectives, AO: >50 μg/L (Health Canada 2017a)

Current AO: >20 μg/L (Health Canada 2019b)

In total, 55 households in the Boreal Plains, Montane Cordillera, Taiga Shield, Boreal Shield, Prairies, Mixedwood Plains, and Atlantic Maritime ecozones had first draw samples greater than 120 μg/L. Sixty flushed samples in these ecozones exceeded the guideline, as shown in Table 8. For comparison, in the Saskatchewan provincial monitoring, 4024 samples were collected from 2003 to 2014 with a mean of 200.5 μg/L and with a maximum of 8404 μg/L (Health Canada 2019c). Based on the FNFNES results, the maximum manganese level was 1530 μg/L in Saskatchewan. The manganese exceedances by ecozone are presented in Appendix 3 and Table 8 and were published in Chan et al. (2019).

Overall, 96 first draw samples had levels of manganese above the AO of 50 μg/L and 109 flushed samples exceeded the guideline value. However, based on the new AO of 20 μg/L, an additional 74 first draw samples and 85 flushed samples were above the AO. When using the previous AO of 50 μg/L, there were 7.2% of flushed samples exceedances compared with 12.8% exceedances, based on the 20 μg/L guideline. Hence, the new AO almost doubles the exceedances of the manganese guideline. The FNFNES results by ecozone are presented in Appendix 3 and Table 8.

The CNDWS (2009–2010) measured manganese concentrations in drinking water at 65 sites across Canada. The average concentrations were 16.1 μg/L in 2009 and 10.8 μg/L in 2010 with 75% of the samples below 11 μg/L in 2009 and below 8 μg/L in 2010. The maximum value was 440 μg/L in 2009 and 160.0 μg/L in 2010 (Health Canada 2019c).

Four percent (60 of 1516 households) of the manganese samples exceeded the MAC and 9.6% (145 of 1516) exceeded the AO. The flushed samples had very similar levels to the first draw samples demonstrating that the source water had elevated manganese levels. In a Manitoba community where all the households had manganese levels above the MAC, the water treatment plant was the source of the elevated manganese. When sampled again 2 months later, the manganese levels in the water treatment plant had returned to acceptable levels.

#### Iron

The Canadian guideline for iron in drinking water remains at 300 μg/L as an aesthetic objective (Health Canada 1987a, 2019b). In total, 56 first draw samples were elevated with values greater than 300 μg/L. Flushing did not lower the iron levels appreciably, with 53 flushed samples above the AO in the Boreal Plains. The summary of the iron results is shown in Table 9.

**Table 9:** Iron concentrations in drinking water by ecozone

Ecozone	No. of first draw samples >300 μg/L	No. of flushed samples >300 μg/L	Maximum concentration, μg/L
Boreal Cordillera	0	0	85
Boreal Plains	11	9	5550
Montane Cordillera	1	2	1420
Pacific Maritime	2	2	1310
Taiga Plains	0	0	76
Taiga Shield	6	10	768
Boreal Shield	26	23	1830
Prairies	2	0	580
Hudson Plains	0	0	0
Mixedwood Plains	7	6	5070
Atlantic Maritime	1	1	589
Total	56	53	5550

Aesthetic objectives, AO: >300 μg/L (Health Canada 2019b)

Data on the concentrations of iron in Canadian drinking water are limited. However, previous data indicated that iron concentrations in drinking water are usually below 300 μg/L. In 1985, the mean iron concentration of raw water sampled in 17 Ontario aquifers was 339 μg/L, whereas treated water had a mean concentration of 46 μg/L (Health Canada 1987a; Health Canada 2009b).

#### Sodium

The Canadian drinking water guideline for sodium has remained as an AO at 200,000 μg/L since 1979 (Health Canada 1992). In total, 77 households in Boreal Plains (with a maximum first draw of 485,000 μg/L), Montane Cordillera (with a maximum first draw of 298,000 μg/L), Prairies (with a maximum first draw of 766,000 μg/L), and Mixedwood Plains (with a maximum of 866,000 μg/L) exceeded the AO value as shown in Table 10.

**Table 10:** Sodium concentrations in drinking water by ecozone

Ecozone	No. of first draw samples >200,000 μg/L	No. of flushed samples >200,000 μg/L	Maximum concentration, μg/L
Boreal Cordillera	0	0	25,600
Boreal Plains	32	33	485,000
Montane Cordillera	1	1	298,000
Pacific Maritime	0	0	62,300
Taiga Plains	0	0	14,700
Taiga Shield	0	0	17,500
Boreal Shield	0	0	125,000
Prairies	26	31	766,000
Hudson Plains	0	0	24,200
Mixedwood Plains	12	12	866,000
Atlantic Maritime	0	0	133,000
Total	71	77	866,000

Aesthetic objectives, AO: >200,000 μg/L (Health Canada 2019b)

The 1980s nation-wide survey of sodium in drinking water supplying 122 municipalities, or 36% of the Canadian population, found that drinking water at the consumer’s tap contained approximately 5.6 mg/L of sodium, as a national median, with concentrations ranging from 0.3 to 242 mg/L (Health Canada 1992). In the FNFNES, most ecozones had sodium levels below the AO. However, in the four ecozones that exceeded the AO, sodium levels were much higher than the values found in the national survey.

#### Zinc

The Canadian drinking water guideline for zinc was developed in 1979 and reviewed in 2005 (Health Canada 2019b). The AO remained at 5000 μg/L. Two households in the FNFNES had elevated zinc in the first draw samples: one in the Boreal Plains with a value of 6890 μg/L and the other in the Boreal Shield with a value of 5150 μg/L (Table 11).

**Table 11:** Zinc concentrations in drinking water by ecozone

Ecozone	No. of first draw samples >5000 μg/L	No. of flushed samples >5000 μg/L	Maximum concentration, μg/L
Boreal Cordillera	0	0	175
Boreal Plains	1	0	6890
Montane Cordillera	0	0	1130
Pacific Maritime	0	0	725
Taiga Plains	0	0	745
Taiga Shield	0	0	2030
Boreal Shield	1	0	5150
Prairies	0	0	2420
Hudson Plains	0	0	3930
Mixedwood Plains	0	0	2760
Atlantic Maritime	0	0	2100
Total	2	0	6890

Aesthetic objectives, AO: >5000 μg/L (Health Canada 2019b)

In the 1981 National Survey of Canadian drinking water supplies, it was found that the median zinc content in the raw, treated, and distributed water samples rarely exceeded 10 μg/L. However, in the 1986 Ontario city survey, where zinc in water was stagnant overnight, levels were up to 100 times greater than in the treated water. In this survey, the concentration of zinc after treatment was less than 10 μg/L, whereas the mean concentration at seven stations following an overnight stand was 309 μg/L (range: 30 to 1170 μg/L). Zinc had been leached from the householders’ pipes when left standing overnight (Health Canada 1987b)

## Conclusion and recommendations

This study provides, for the first time in a peer-reviewed manuscript, a snapshot of the levels of metals typically found in drinking waters in First Nations across the country. Overall, the quality of drinking water regarding the trace metal levels is satisfactory with some exceptions. Our results show that the metal exceedance can be caused by either the piping within the households or the water supply system in the communities. For example, elevated lead and copper in households, where exceedances of the MAC were much greater in the first draws than in the flushed samples, resulted from the households’ pipes. Whereas, for manganese, uranium, aluminum, iron, and sodium, exceedances of the guidelines were attributed to elevated levels in the community’s distribution system.

All exceedances were communicated to environmental public health professionals at the First Nations and Inuit Health Branch shortly after the analyses to ensure that necessary mitigation actions may take place, as per study design. First Nations received community reports upon the completion of each regional study. With the revised guideline of 5 μg/L in a first draw water sample for lead, 8.4% (128 of 1516) of households exceed the new guideline. Until appropriate corrective actions are in place, a 5-min flush would lower the lead in drinking water to acceptable levels in more than 90% of the households that participated in the study. An alternative approach to minimize exposure to lead could be the implementation of drinking water treatment devices. It would be interesting to repeat this study in the future with the present Health Canada sampling methodology. Similarly, copper has a new health guideline of 2000 μg/L. Flushing water for 5 min removes 80% of the copper exceedances. Therefore, flushing is also recommended to reduce most of the elevated copper levels. The levels of metals in drinking water systems can also be controlled by the use of corrosion inhibitors as well as pH or alkalinity adjustments.

Other issues related to the quality of drinking water identified by the study participants were usually associated with the aesthetic or taste of the waters. It would be of interest to examine in future research the associations between rates of dissatisfaction with the organoleptic quality of drinking water and rates of exceedances of the criteria established for those parameters. Regular maintenance and improvement of the water treatment and delivery system need to be implemented to improve the quality of the drinking water supply and increase acceptance and confidence within the population, reducing the use of onerous and often unecological water sources such as bottled water. The research team has been working with the First Nations and the regional environmental health officers on the knowledge translation of the results to ensure that First Nations are provided with a safe and pleasant drinking water supply.

## Appendix 1

### Aluminum

#### Exceedances of an OG of 100 and 50 μg/L by ecozone (Chan et al. 2019)

##### Boreal Plains

Twenty households in one Alberta Boreal Plains community exceeded the OG with levels ranging from 111 to 621 μg/L. Two households in two Saskatchewan Boreal Plains communities exceeded the OG with values ranging from 153 to196 μg/L. Also, 20 households in one Manitoba Boreal Plains community exceeded the OG with values from 110 to 142 μg/L. An additional 6 first draw samples and 2 flushed samples exceeded the proposed guideline of 50 μg/L.

##### Montane Cordillera

In total, 11 households in one British Columbia Montane Cordillera community exceeded the OG with values ranging from 140 to 287 μg/L.

##### Taiga Shield

Fifteen households in one Manitoba Taiga Shield community exceeded the OG with values ranging from 431 to 1060 μg/L.

##### Boreal Shield

Seven households in one Saskatchewan Boreal Shield community exceeded the OG with values ranging from 105 to 144 μg/L in flushed samples. Eleven households in two Manitoba Boreal Shield communities exceeded the OG with values ranging from 102 to 747 μg/L. Seventeen households in three Ontario Boreal Shield communities exceeded the OG with values ranging from 101 to 512 μg/L. Two households in one Quebec Boreal Shield community exceeded the OG with values ranging from 105 to 157 μg/L. Also, 21 households in one Atlantic Boreal Shield community exceeded the OG with values ranging from 108 to 806 μg/L. When considering the proposed guideline of 50 μg/L, an extra 38 flushed samples exceeded the guideline value.

##### Prairies

Sixteen households in one Manitoba Prairies community exceeded the OG with values ranging from 101 to 290 μg/L. An additional 2 households had aluminum levels in flushed samples ranging from 50 to 99 μg/L.

##### Hudson Plains

Twenty-one households in two Ontario Hudson Plains communities exceeded the OG with values ranging from 127 to 1920 μg/L. One household had an aluminum level within the range of 50 to 99 μg/L.

##### Mixedwood Plains

Eleven households in two Ontario Mixedwood Plains communities exceeded the OG with values ranging from 105 to 596 μg/L. Also, 14 additional flushed water samples exceeded the proposed guideline of 50 μg/L.

##### Atlantic Maritime

Twenty-one households in three Atlantic Maritime communities exceeded the OG for aluminum with values ranging from 103 to 543 μg/L. Also, two additional households exceeded the proposed guideline of 50 μg/L.

## Appendix 2

### Copper

#### Exceedance of the MAC level of 2000 μg/L by ecozone (Chan et al. 2019)

##### Boreal Plains

One Saskatchewan Boreal Plains household had a first draw sample of 5130 μg/L while a flushed sample was below the proposed MAC of 2000 μg/L.

##### Montane Cordillera

One British Columbia Montane Cordillera household had a first draw sample of 2000 μg/L.

##### Pacific Maritime

Four British Columbia households in two Pacific Maritime communities had first draw samples ranging from 2380 to 2930 μg/L. However, all flushed samples were below the proposed MAC of 2000 μg/L.

##### Boreal Shield

Three households in one Manitoba Boreal Shield community had first draw samples ranging from 2290 to 4240 μg/L. Also, two households in one Atlantic Boreal Shield community had first draw samples ranging from 2170 to 2260 μg/L. After flushing, the copper levels were below the proposed guideline of 2000 μg/L. After a 5-min flush, only two households had elevated copper levels ranging from 3950 to 6540 μg/L.

##### Hudson Plains

Three households in two Ontario Hudson Plains communities had first draw samples ranging from 2050 to 3460 μg/L, while all flushed samples were below the MAC.

##### Mixedwood Plains

One Ontario Mixedwood Plains household had a first draw sample of 5850 μg/L and a flushed sample of 3380 μg/L which exceeded the new guideline value as shown in Table 7.

#### Exceedances of the AO of 1000 μg/L by ecozone

##### Boreal Plains

One household in a British Columbia Boreal Plains community had a first draw sample of 1170 μg/L. Also, four households in four Saskatchewan Boreal Plains communities had first draw samples ranging from 1170 to 1700 μg/L. In addition, three households in one Manitoba Boreal Shield community had first draw samples ranging from 1020 to 1820 μg/L. All flushed samples were below 1000 μg/L.

##### Montane Cordillera

One British Columbia Montane Cordillera community had a first draw sample of 1340 μg/L.

##### Pacific Maritime

Nine households in three British Columbia Pacific Maritime communities had first draw samples ranging from 1020 to 1910 μg/L. All flushed samples were below the AO of 1000 μg/L.

##### Taiga Shield

One household in one Manitoba Taiga Shield community had a first draw sample of 1260 μg/L. Also, one household in one Quebec Taiga Shield community had a first draw sample of 1270 μg/L. The flushed samples had copper levels below 1000 μg/L.

##### Boreal Shield

Five households in two Manitoba Boreal Shield communities had first draw samples ranging from 1060 to 1490 μg/L. Seven households in two Ontario Boreal Shield communities had first draw samples ranging from 1030 to 1680 μg/L. Nine Atlantic Boreal Shield households in one community had first draw samples ranging from 1060 to 1850 μg/L. After a 5-min flush, all samples were below the AO of 1000 μg/L.

##### Prairies

One household in one Saskatchewan Prairies community had a first draw sample of 1260 μg/L. The flushed sample for this household remained elevated at 1190 μg/L. Also, one Manitoba Prairies household had a first draw sample of 1890 μg/L and a flushed sample below 1000 μg/L.

##### Hudson Plains

Five households in three Ontario Hudson Plains communities had first draw samples ranging from 1030 to 1690 μg/L and all flushed samples were below the AO.

##### Mixedwood Plains

Four households in three Ontario Mixedwood Plains communities had first draw samples ranging from 1080 to 1630 μg/L. Two flushed samples in two of the Ontario communities had values ranging from 1300 to 1350 μg/L.

##### Atlantic Maritime

Three households in two Atlantic Maritime communities had first draw samples ranging from 1070 to 1570 μg/L. However, all flushed samples were below 1000 μg/L.

## Appendix 3

### Manganese

#### Exceedance of the MAC of 120 μg/L by ecozone (Chan et al. 2019)

##### Boreal Plains

Two households in the Boreal Plains ecozone in two communities in Saskatchewan had first draw samples ranging from 127 to 175 μg/L. The 5-min flush samples remained elevated with levels ranging from 131 to 157 μg/L. Also, one community in the Boreal Plains ecozone in Manitoba had a flushed sample exceeding the MAC with a level of 191 μg/L.

##### Montane Cordillera

Two households in one community in the Montane Cordillera ecozone in British Columbia had first draw samples ranging from 126 to 164 μg/L. The flushed samples exceeded the MAC with values ranging from 127 to 250 μg/L.

##### Taiga Shield

Two households in the Taiga Shield ecozone in one community in Saskatchewan had first draw samples ranging from 121 to 124 μg/L. Six flushed samples exceeded the MAC with values ranging from 124 to 142 μg/L.

##### Boreal Shield

Twenty households in the Boreal Shield ecozone in one community in Manitoba had first draw samples ranging from 231 to 444 μg/L. The flushed samples exceeded the MAC with values ranging from 228 to 321 μg/L.

##### Prairies

Five households in the Prairies ecozone in two communities in Saskatchewan had first draw samples ranging from 120 to 1530 μg/L. The 5-min flush samples exceeded the MAC with values ranging from 150 to 1520 μg/L.

##### Mixedwood Plains

One household in one Quebec Mixedwood Plains community had a first draw sample of 370 μg/L. After a 5-min flush, the sample exceeded the MAC with a value of 361 μg/L.

##### Atlantic Maritime

One Quebec Atlantic Maritime household had a first draw sample of 975 μg/L. The flushed sample exceeded the MAC guideline with a value of 929 μg/L. In addition, 22 households in four Atlantic Maritime communities had first draw samples ranging from 127 to 532 μg/L. After a 5-min flush, all households exceeded the MAC with values of 141 to 685 μg/L.

#### Exceedance of the AO of 50 and 20 μg/L by ecozone

##### Boreal Cordillera

One household in one British Columbia Boreal Cordillera community had a first draw sample of 69.8 μg/L. After a five-minute flush, the value exceeded the previous AO with a value of 68 μg/L. Four first draw samples in this community were in the range from 20 to 49 μg/L. Five additional flushed samples exceeded the new AO with values ranging from 20.9 to 31.6 μg/L.

##### Boreal Plains

One household in one British Columbia Boreal Plains community had a first draw value of 77.1 μg/L. After a 5-min flush, the AO was exceeded with a value of 67.6 μg/L.

Six households in four Alberta Boreal Plains communities had first draw values ranging from 50.4 to 86.3 μg/L. After flushing, six households in two of the communities exceeded the previous AO with levels ranging from 50.9 to 87.5 μg/L. In addition, four first draw households in three Alberta Boreal Plains communities were in the range from 20 to 49 μg/L with values from 29.2 to 39.6 μg/L. After a 5-min flush, there were an additional 11 households in two Alberta communities that exceeded the 20 μg/L AO manganese guideline.

Three households in three Saskatchewan Boreal Plains communities had first draw samples of greater than 50 and less than 120 μg/L with values ranging from 58.3 to 100 μg/L. After flushing, two households in two of these communities had values from 92.7 to 117 μg/L. In addition, four households in three Saskatchewan Boreal Plains communities had first draw values in the range from 20 to 49 μg/L with values ranging from 21.1 to 44.2 μg/L. After flushing, the same households exceeded the new AO with levels ranging from 33.7 to 43.9 μg/L.

Four Manitoba Boreal Plains households in two communities had values greater than the new AO with values ranging from 22.1 to 27.3 μg/L. After flushing, only one household exceeded the AO with a value of 24.9 μg/L.

##### Montane Cordillera

Two households in one British Columbia Montane Cordillera community had first draw samples greater than 50 and less than 120 μg/L with values 83 to 87 μg/L. After a 5-min flush, one household in this community had a value of 109 μg/L. Also, one household in the community above had a first draw value of 48.1 in the range from 20 to 49 μg/L. An additional four 5-min flush samples in two British Columbia Montane Cordillera communities exceeded the manganese AO with values ranging from 21.5 to 47.6 μg/L.

##### Pacific Maritime

One household in one British Columbia Pacific Maritime community had a first draw sample of 38.8 μg/L. After flushing, this household exceeded the manganese AO with a value of 44.4 μg/L.

##### Taiga Plains

One British Columbia Taiga Plains household had a first draw value of 20.6 μg/L.

##### Taiga Shield

Five Saskatchewan Taiga Shield households had first draw samples ranging from 51.1 to 88.1 μg/L. After flushing, eight households had manganese exceeding the previous AO with values of 54.8 to 117 μg/L. In addition, seven households in this community had first draw samples between 20 and 49 μg/L ranging from 22.0 to 48.5 μg/L. Among those, three flushed samples exceeded the new guideline with values ranging from 41.2 to 49.3 μg/L.

##### Boreal Shield

One flushed sample in one Ontario Boreal Shield community had a value greater than 50 and less than 120 μg/L with a value of 78.8 μg/L. Also, two households in the above community had first draw samples ranging from 25.3 to 37.1 μg/L. After flushing, four households in two Ontario Boreal Shield communities exceeded the new manganese AO with values ranging from 20.1 to 28.8 μg/L.

##### Prairies

One household in one Alberta Prairies community had a first draw of 80.4 μg/L in the range greater than 50 and less than 120 μg/L. Three households in this community had a first draw between 20 and 49 μg/L with a range from 30.9 to 39.2 μg/L while seven flushed samples exceeded the new manganese AO with values ranging from 21.1 to 38.3 μg/L.

Three households in one Saskatchewan Prairies community had a first draw with values greater than 50 and less than 120 μg/L ranging from 64.2 to 83.1 μg/L. Five 5-min flush samples in this community exceeded the previous AO with values ranging from 79.7 to 111 μg/L. Also, seven households in three Saskatchewan Prairies communities had first draw samples between 20 and 49 μg/L with a range from 21.0 to 47.8 μg/L. After a 5-min flush, six households exceeded the new AO with a range of 22.7 to 42.6 μg/L.

Five households in one Manitoba Prairies community had first draw samples greater than 50 and less than 120 μg/L with a range of 51.1 to 68.7 μg/L. Six flushed samples from this community exceeded the previous AO with values ranging from 56.5 to 80.5 μg/L. In addition, seven households in two Manitoba Prairies communities had first draw samples from 20.1 to 38.8 μg/L and seven 5-min flush samples exceeded the new AO of 20 μg/L.

##### Hudson Plains

Five flushed samples in one Ontario Hudson Plains community exceeded the previous AO of 50 μg/L with values ranging from 50.0 to 61.5 μg/L. Also, two households in this same community had first draw samples between 20 and 49 μg/L with a range of 48.7 to 49.3 μg/L. After a 5-min flush, four additional samples exceeded the new AO with a range of 37.9 to 45.7 μg/L.

##### Mixedwood Plains

Four households in two Ontario Mixedwood Plains communities had first draw samples greater than 50 and less than 120 μg/L with a range of 51.5 to 115 μg/L. After flushing, five samples in these two communities exceeded the previous AO with a range of 52.5 to 96 μg/L. In addition, nine households in three Ontario Mixedwood Plains communities had first draw samples between 20 and 49 μg/L with a range of 21.1 to 43.8 μg/L. After flushing, five households exceeded the new AO with levels from 23.1 to 40.5 μg/L. Finally, one Quebec Mixedwood Plains community had a first draw sample of 27.8 μg/L. After flushing, that household had a value of 24.4 μg/L.

##### Atlantic Maritime

Ten households in three Atlantic Maritime communities had first draw samples greater than 50 and less than 120 μg/L with a range of 50.8 to 119 μg/L. After flushing, eight households in these three communities exceeded 50 μg/L and were lower than the MAC with a range of 53.2 to 99.5 μg/L. In addition, 17 households in four Atlantic Maritime communities had first draw samples in the 20 to 49 μg/L range of 21.2 to 46.3 μg/L. After flushing, 21 additional samples exceeded the new AO with a range of 22.5 to 46.2 μg/L. Also, one flush sample in the Atlantic Maritime ecozone in Quebec exceeded the new AO with a value of 20.0 μg/L.

## Appendix 4

**Table 12:** Tap water sample collection for metals of human health concern and metals that have operational guidance values and aesthetic objectives, by ecozone

Years sampled	Ecozone	No. of communities	No. of households invited to participate	No. of households that participated	Participation rate, %	No. of first draw samples	No. of flushed samples	No. of duplicate samples	Total no. of samples
2008	Boreal Cordillera	2	41	34	82.9	34	34	8	76
2009, 2010 2013, 2015	Boreal Plains	17	341	250	73.8	242	238	54	534
2008–2009	Montane Cordillera	6	123	94	76.4	93	92	17	202
2008–2009	Pacific Maritime	9	185	151	81.6	138	139	32	309
2008, 2013	Taiga Plains	2	40	18	45.0	18	18	7	43
2010, 2015, 2016	Taiga Shield	5	100	60	60.0	53	60	14	127
2010, 2011 2012, 2015	Boreal Shield	19	380	291	76.6	282	288	71	641
2010, 2013, 2015	Prairies	8	160	131	81.9	124	131	21	276
2011, 2012, 2016	Hudson Plains	5	100	78	78.0	67	75	19	161
2012, 2016	Mixedwood Plains	6	180	173	96.1	172	166	67	405
2014, 2016	Atlantic Maritime	12	243	236	97.1	232	237	46	515
	Total	91	1893	1516	80.1	1455	1478	356	3289
